# Assessment of Environmental Impacts on Health: Examples from the Pacific Basin

**DOI:** 10.5334/aogh.3671

**Published:** 2022-10-21

**Authors:** Paul Jagals, Injeong Kim, Claire Brereton, Colleen L. Lau

**Affiliations:** 1 Children’s Health and Environment Program, Child Health Research Centre, The University of Queensland, Australia; 2 Korea Institute of Industrial Technology, Seoul, South Korea; 3 School of Public Health, The University of Queensland, Australia

**Keywords:** Environmental Health Risk and Impact Assessment, System Dynamics, Causal loop diagrams, Precision Environmental Public Health

## Abstract

Assessing environmental impacts on health in the Pacific Basin is challenged by significantly varying data types – quantities, qualities, and paucities – because of varying geographic sizes, environments, biodiversity, ecological assets, and human population densities, with highly varied and unequal socio-economic development and capacity to respond to environmental and health challenges. We discuss three case-based methodological examples from Pacific Basin environmental health impact assessments. These methods could be used to improve environmental health evidence at all country and regional levels across a spectrum of big data availability to no data. These methods are, 1) a risk assessment of airborne particulate matter in Korea based on the chemical composition of these particulates; 2) the use of system dynamics to appraise the influences of a range of environmental health determinants on child health outcomes in remote Solomon Islands; and 3) precision environmental public health methodologies based on comprehensive data collection, analyses, and modelling (including Bayesian belief networks and spatial epidemiology) increasing precision for good environmental health decision making to prevent and control a zoonotic disease in Fiji Islands. We show that while a common theme across the three examples is the value of high quality and quantity data to support stronger policy decisions and appropriate prioritizing of investment, it is also clear that for many countries in the Pacific Basin, sufficient data will remain a challenge to inform decision makers about environmental impact on health.

## Introduction

The impacts of environmental conditions, agents, and states on health (we also refer to this as environmental health), are often easy to conceptualise (environmental determinants of health). It generally is, however, much more difficult to find sufficient environmental health evidence to sway societies into action to prevent environmental hazards and exposures as well as promote environmental health. This challenge results from our limited abilities to make sense of complex relationships in and between the major domains of human society – summarised in health, environment, social, economic, and technical (HESET) domains [1].

Half of the world’s population live in countries that have a direct proximity to the Pacific Ocean (commonly referred to as the Pacific Basin). These countries and their societies vary significantly across spectrums of geographic sizes, environments, biodiversity, ecological assets, culture, lifestyle, and human population densities, with highly varied and unequal socio-economic development and capacity to respond to environment and health challenges [2].

Environmental determinants of health for these populations are therefore very heterogenous and not evenly distributed, most often not comprehensively measured and modelled, and not managed according to a common blueprint to ensure healthy environments for healthy people in the region [3,4]. Large-scale environmental conditions and states such as natural disasters and climate change have globally significant and visible environmental influences and impacts on health. These conditions receive advocacy and attention at broader and higher levels and are therefore generally better understood. There are, however, more subtle and local environmental states, conditions, and agents driving the occurrence of infectious and non-communicable diseases. These drivers are often misunderstood and often underestimated, leading to societal actions that are more likely to exacerbate these negative environmental influences than prevent them.

Examples of local environmental agents, conditions, and states are health-related air quality as well as water/food safety and security. These are not comprehensively and equally measured in the context of potential human health impact across Pacific Basin countries. Some countries collect large quantities of high-quality data which enables analyses, interpretation, and good evidence syntheses for sound decision-making on environmental health actions [5]. For most Pacific Basin countries – especially the small island states of the Pacific – a paucity of data and limited understanding of the impacts and unintended consequences of actions can lead to poor-quality decision-making about environmentally-protective socio-economic development [1,6,7]. Assessment methods are called for that can accommodate good, as well as limited data drawn from multiple (HESET) sectors of society.

In this report, we discuss three case-based methodological examples from the Pacific Basin to assess environmental impacts on health. These methods could be used to improve environmental health evidence at all country and regional levels across a spectrum of availability of big data to sparse data. Firstly, we present a health risk assessment based on the chemical composition of airborne particulate matter in Korea to provide a clearer picture of health risk posed by such an environmental agent – in this example – airborne particulate matter. Secondly, we demonstrate the use of systems science methods that offer the potential to combine many different and heterogenous sources, levels, and types of data as well as options for incorporating data based on knowledge, opinions, and judgements in situations where empirical data are scarce. We present an example of the use of system dynamics to appraise the influences of a range of environmental health determinants on child health outcomes in remote Solomon Islands. Finally, using an example of prevention and control of a zoonotic disease (Leptospirosis) in Fiji, we discuss the concept of precision environmental public health to show how comprehensive data collection (again – from heterogenous sources, levels, and types), analyses, and modelling (including Bayesian belief networks and spatial epidemiology) could increase the precision of good environmental health decision making.

## Assessment of Health Risk Posed by Airborne Ultrafine Particles in an Urban Environment

Health-related air quality has long been a problem for populations in countries in the proximity of the Pacific Basin [8]. More recently, an acute awareness is also developing about the impact of air quality on the health of people in the more remote Pacific Island Countries and Territories [9]. A popular indicator of air quality is mass-based concentrations of health-relevant particulates in the ambient and household air. In larger Pacific Basin countries, with the capabilities to measure air quality on a large scale, epidemiological assessment using big data sets have provided evidence that respiratory tract infections are linked to exposure to poor-quality air. For the Pacific Island Countries and Territories, large scale measurement of air quality is not yet an established environmental public health practice [9,10].

**Figure 1:**
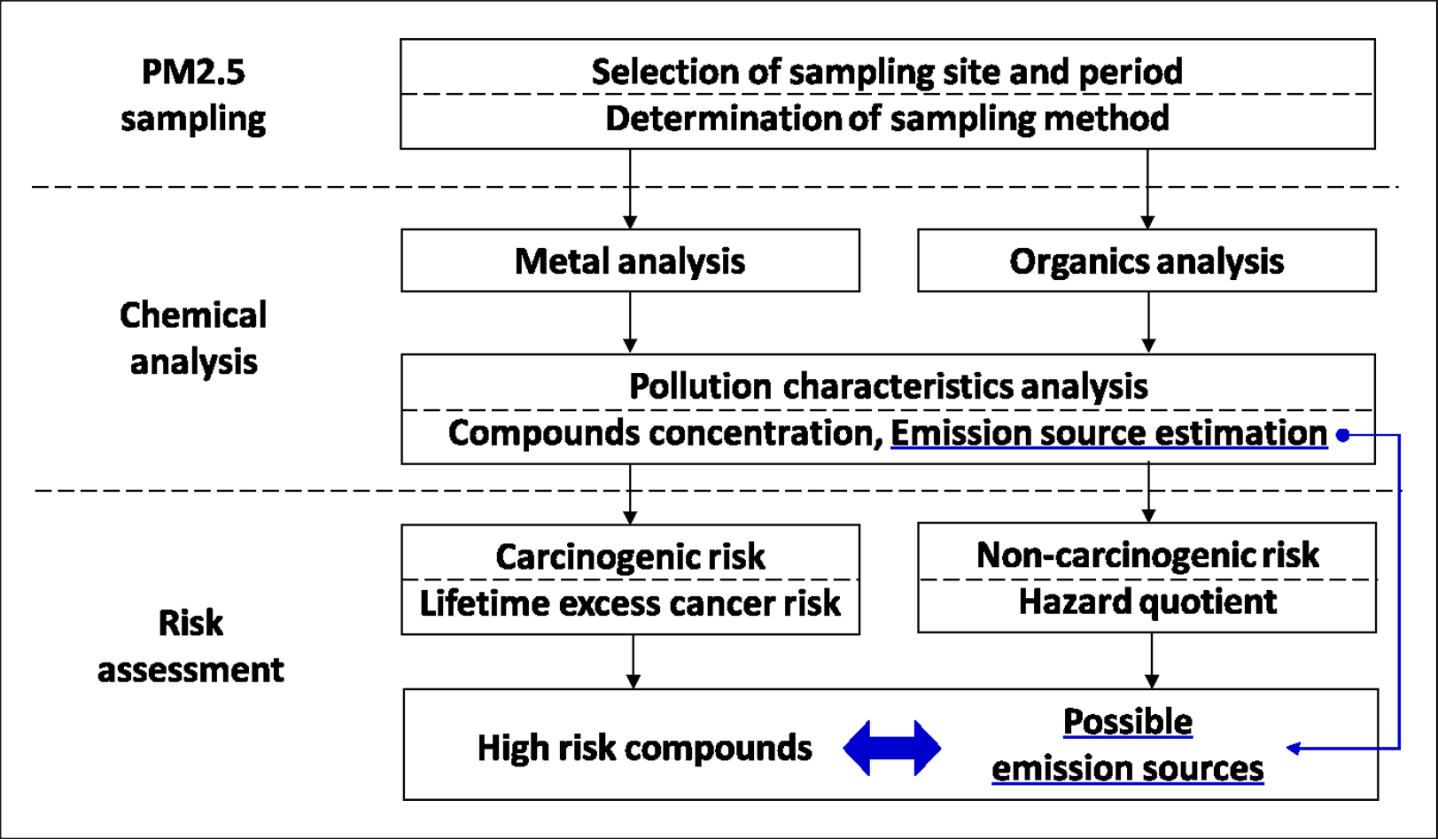
Flow chart of PM_2.5_ Health Risk Assessment.

Furthermore, getting proper insights into the health significance of ambient and indoor particulate matter especially requires more than just measuring its presence in the air. Increasing evidence suggests that the toxicity of fine dust particles (PM_2.5_) is linked to specific components of the particles rather than their mass [11]. However, our understanding of the chemical composition of PM_2.5_ and the health risk this poses is currently limited. Particles in air, especially those released from combustion processes (industry, energy generation, motorised vehicles), contain metals, polycyclic aromatic hydrocarbons (PAHs), organochlorine pesticides (OCPs), and polychlorinated biphenyls (PCBs) amongst others – all of which can be detrimental to health if present in hazardous concentrations in inhaled air. These particles can increase the risk of disease such as respiratory infections and cancer. Kim et al. [11] used a toxicologic risk assessment approach to understand these component parts within the particles to determine the health risks posed by PM_2.5_ (Figure 1).

A Health Risk Assessment (HRA) approach was used to structure the assessment of risk posed by exposure to the hazardous components of the PM_2.5_ airborne particulates sampled for this study. HRA provides the scientific evidence for the development of risk management plans [12].

To assess health risk posed by airborne PM_2.5_, data about the concentration of toxic compounds in the particles and their toxicity value are essential to determine the likely response to the dose of these substances during exposure. Whilst toxicity information for a substance is readily available through several databases such as Integrated Risk Information System (USEPA) [13], the identification and quantification of the toxic compounds within PM_2.5_ particles require scientific effort (monitoring/sampling, chemical analysis etc.). For example, Kim et al. [11] conducted PM_2.5_ sampling for 21 weeks in Gwangju, Korea, and analysed the concentration of 20 metals and 114 organics in the sampled particles. The data were used to assess risk posed by PM_2.5_, as well as apportion the sources of PM_2.5_ to provide insights as to the likely sources of pollution. Following these scientific processes, they suggested that Mn, BaP, Pb, As, and Cd contribute most to the health risk posed by the particles. They also provided the scientific evidence to target the emission sources that should be regulated to reduce the PM_2.5_ risk. In the case of Gwangju, Korea, the emission reduction from industrial activities, oil combustion, and gasoline exhaust were proposed to be most effective in terms of risk reduction compared to other emission sources. Air pollutants are emitted through various emission sources, and it might be socio-economically impossible to regulate all sources. The evidence provided by studies like this can guide decision makers in the management of emission sources by prioritizing them based on human health risk, and effectively provide large social benefits at a lower cost. This approach can also provide useful information for other environmental states, agents, and conditions to decision makers for effective management of environmental health risk [14].

## System Science

Environmental health can be viewed as a complex system, with interconnected elements which interact to produce non-linear effects, which are different from the mostly linear effects of the individual elements. Systems science, the application of scientific methods to understand complex systems, provides insights into how these systems work [1,15]. We use its structured methods to explore and understand how component parts of the environmental health system connect and interact, thus increasing our capability to understand complex problems, provide evidence, and design workable solutions. Causal assessment of environmental conditions, agents and states, socio-economic factors, and their relationship with, and/or risks to health and wellbeing is hampered by a paucity of data. System dynamics is a systems science approach which can be applied to partially compensate for this, blending available data with expert opinion [15].

Solomon Islands is a remote and extremely environmentally vulnerable Least Developed Country in the South Pacific Ocean of the Pacific Basin. The country faces challenges of poverty, population growth, rapid urbanisation with lagging infrastructure development, increasing pollution, and climate change. Forty percent of the population is under 16 years old [16]. We used a system dynamics approach [17] in Solomon Islands to identify cause-effect relationships linking a selected range of environmental determinants of children’s health outcomes, and then to simulate different scenarios and quantify the effects of potential interventions designed to improve those outcomes.

**Figure 2:**
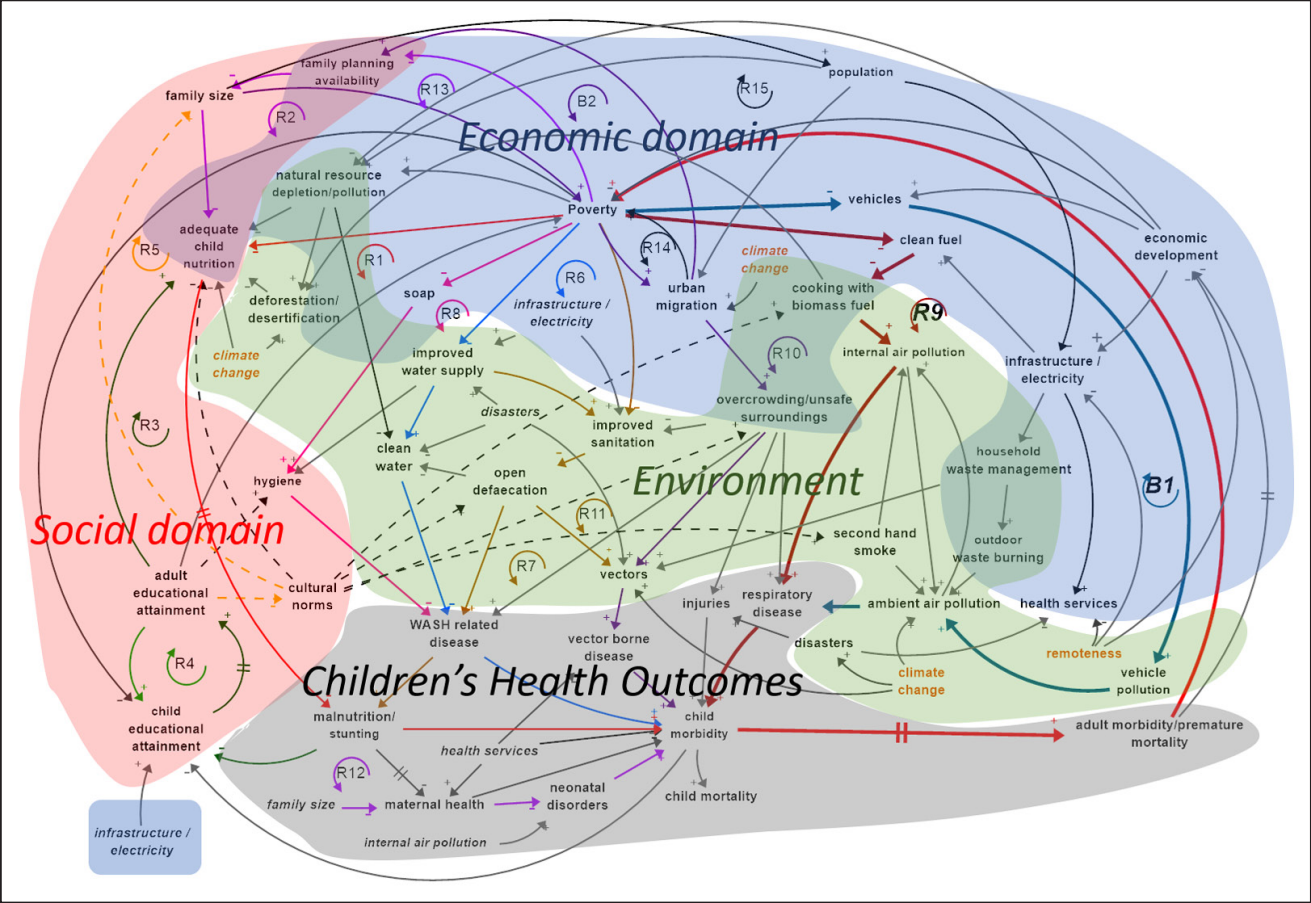
Causal Loop Diagram – Environmental, economic and social domains intersecting and reinforcing to influence children’s health outcomes in Solomon Islands [1].

Firstly, a causal loop diagram was developed (Figure 2) showing significant causal associations and identifying feedback loops. Feedback, or circular causality, occurs when a set of causal associations connect to make a closed path. Feedback loops are either reinforcing, generating exponential growth or decline over time, or balancing, where one effect counteracts another to reach an equilibrium. Most feedback loops in this diagram make links between children’s health outcomes and multiple HESET domains and most, denoted by the “R” symbols, are reinforcing.

Secondly, a quantitative dynamic model was built – using the system structure defined by the causal loop diagram – to simulate different scenarios (Figure 2). Simulations included investments in areas such as water and sanitation infrastructure, education, and family planning. They also included simulation of different global CO_2_ emissions scenarios. The simulations enabled comparison of the projected effects of different socio-economic environmental scenarios on children’s health.

**Figure 3:**
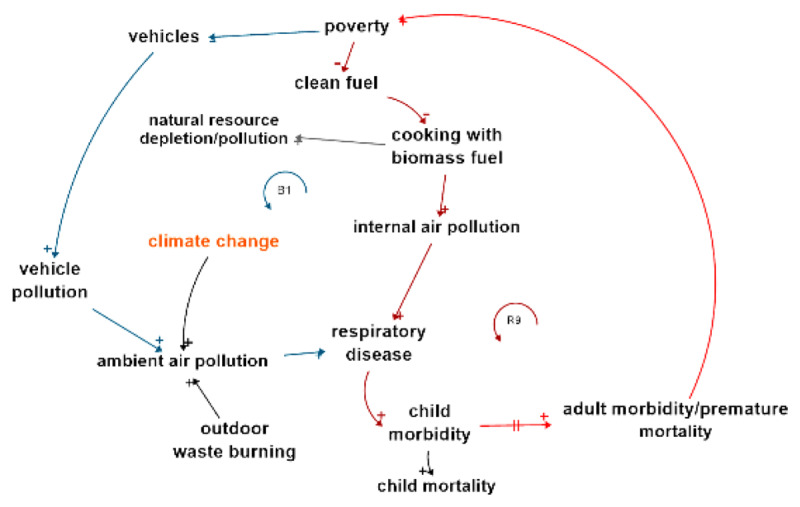
Sample air quality causal loop from Figure 2.

One of the many reinforcing loops in this complex system is the adverse impacts of air quality on health in Solomon Islands. Figure 3 shows reinforcing loop R9 (shown in bold in Figure 2) which illustrates the links between biomass fuel use, child morbidity and mortality, and adult morbidity and premature mortality, which reinforces poverty due to a reduced ability to contribute economically. Poverty in its turn reduces access to clean fuel and reinforces the cycle.

Balancing loop B1, shown in bold in Figure 2, shows that poverty reduces the ability to buy/use vehicles which reduces pollution related to automobile or marine fuel consumption, which reduces ambient air pollution, a cause of respiratory disease. Conversely, reducing poverty increases respiratory disease. These two loops illustrate the complexity of environmental health decisions; poverty is at the root of many of the socio-economic causes of health outcomes, but when economic state improves, new environmental health hazards are created.

Solomon Islands survey data [18] show that 33% of the population have no access to improved water supplies, 67% have no sanitation, 25% have no electricity (90% cook on wood fires), leading to a range of environmental hazards which can impact on health. Availability of basic drinking water services has declined gradually over the last 15 years as earlier water investments have reached the end of their lifespan, exacerbated by increasing demand from a growing population [19]. Simulation modelling shows that population growth is outpacing water and sanitation infrastructure investment and putting pressure on general health and education investment. It highlights priority data gaps such as causes of child mortality which must be addressed to deliver an effectively targeted approach to supporting and protecting children’s health in Solomon Islands. The simulation implies that, based on the trends in the structural causes of children’s morbidity and mortality in Solomon Islands, children’s health is not improving, in contrast to global trends, but there is insufficient data to validate this finding. The model shows that limited access to modern family planning facilities, which are currently available to less than 30% of families [18], is contributing to population growth. Without significantly increased investment in water and sanitation infrastructure in both rural and informal settlements to support a growing population, child mortality rates will not improve in the next 10 years. Even with investment, Solomon Islands is unlikely to meet health related Sustainable Development Goal targets due to the lead times required for health outcomes to improve [20].

## Precision Environmental and Public Health

**Figure 4:**
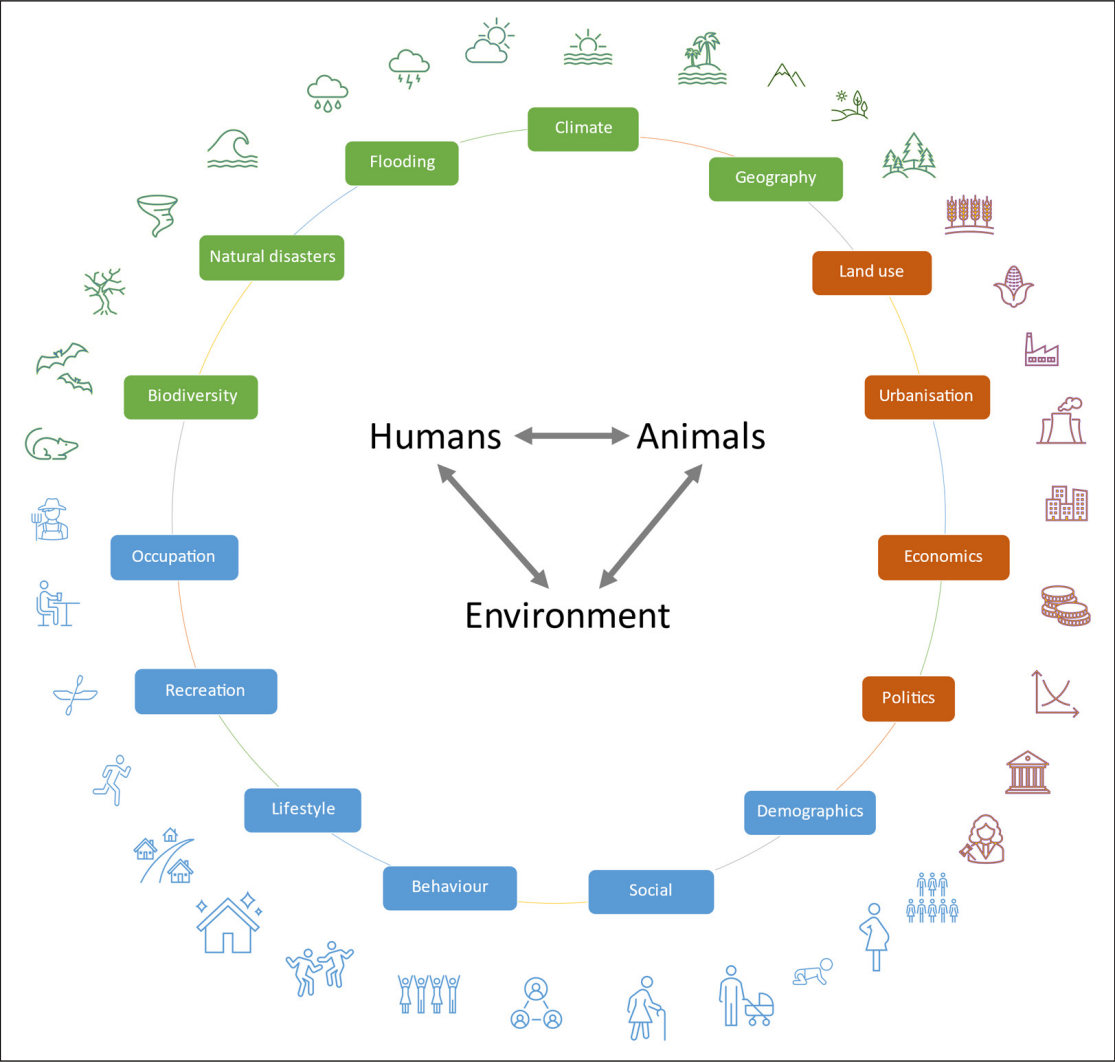
Infectious disease transmission is strong driven by interactions between humans, animals, and the environment. The interactions are in turn influenced by multiple interrelated environmental and sociodemographic factors.

Infectious diseases are responsible for significant disease burden in the Pacific Islands Countries and Territories. Transmission and outbreaks are strongly driven by multiple interrelated environmental and socio-demographic factors (Figure 4), as well as the complex interactions between humans, animals, and vectors. Challenges in the prevention and control of infectious diseases include poor quantification of disease burden, poor evidence about how best to manage social and environmental risk factors and drivers, and limited evidence-based strategies for interventions [21].

Environmental drivers of disease transmission vary between diseases, differ between times of emergence, outbreaks, and during the last stages of elimination programs where prevalence has reached very low levels. Effective disease prevention and control strategies depend critically on efficient data collection and good surveillance systems, so that appropriate interventions can be implemented at the right place and time. Innovative approaches are needed to provide early warning of disease outbreaks, e.g. sentinel surveillance, improved environmental monitoring to provide indicator signals, and the use of geospatial tools to identify and predict hotspots [22].

Precision public health aims to make optimal use of limited public health resources by better *stratification* of populations and diseases, so that more precise interventions can be delivered at the right *time* and right *place* to the right *population* [23]. To achieve these goals, innovative methods and cutting-edge technologies are used to *measure* disease, pathogens, exposures, behaviours, and susceptibility more accurately in populations. This includes improving the volume, variety, timeliness, and validity of *data*. Furthermore, advances in data science and *analytics* are used to improve understanding of disease risk factors and drivers, thereby enabling more precise interventions in terms of time, place, and populations. Models can also help predict the timing and location of disease emergence and outbreaks, enable analysis of complex scenarios, and support evidence-based decision making.

Leptospirosis is a zoonotic disease with strong environmental drivers of transmission and causes very high disease burden in the Pacific Islands [24]. Examples of studies that have provided evidence to improve the precision of prevention and control strategies for leptospirosis in Fiji include improving risk stratification through eco-epidemiological studies [25], Bayesian network modelling [26], and spatial analyses such as geographically weighted regression, spatial Bayesian networks, and predictive risk mapping [27,28,29]. These studies integrated multiple sources of data (including human and animal surveys, census, climate, agriculture) and used a variety of advanced analytic methods to better stratify disease burden and risk factors between subpopulations and identify high risk locations. Knowledge about differences in the risk of leptospirosis between age groups, gender, ethnicity, and occupations enables more specific targeting of prevention and control strategies, including health information messaging. Using a geographically weighted regression model, the studies showed that even in the small islands of Fiji, environmental drivers of leptospirosis transmission varied significantly over space [27] Significant environmental drivers included livestock density, rainfall, and poverty but the relative importance of these drivers varied between regions. Knowledge about these differences allow prioritisation of public health interventions that are most likely to be effective in that region.

## Discussion and Conclusion

A common theme across all three examples in this paper is the value of data. The examples demonstrated a range of environmental health assessment methods that could be applied depending on the quality and quantity of data available – ranging from high resolution data collected to specifically address environmental health questions, to situations where there was little or no data. It is also clear that for many countries in the Pacific Basin, collecting new, especially local data will remain a challenge.

The examples of structured assessment methods (risk assessment, system science, and precision environmental public health) demonstrate that many approaches can be used to improve the knowledge required to make good environmental health decisions, while optimising what data are available. These types of assessments and evidence will support stronger policy decisions and more precise and appropriate prioritisation of environmental health investments.
